# Extended Preclinical Safety, Efficacy and Stability Testing of a Live-attenuated Chikungunya Vaccine Candidate

**DOI:** 10.1371/journal.pntd.0004007

**Published:** 2015-09-04

**Authors:** Kenneth S Plante, Shannan L. Rossi, Nicholas A. Bergren, Robert L. Seymour, Scott C. Weaver

**Affiliations:** Institute for Human Infections and Immunity, Sealy Center for Vaccine Development and Department of Pathology, University of Texas Medical Branch, Galveston, Texas, United States of America; The George Washington University School of Medicine and Health Sciences, UNITED STATES

## Abstract

We recently described a new, live-attenuated vaccine candidate for chikungunya (CHIK) fever, CHIKV/IRES. This vaccine was shown to be well attenuated, immunogenic and efficacious in protecting against CHIK virus (CHIKV) challenge of mice and nonhuman primates. To further evaluate its preclinical safety, we compared CHIKV/IRES distribution and viral loads in interferon-α/β receptor-incompetent A129 mice to another CHIK vaccine candidate, 181/clone25, which proved highly immunogenic but mildly reactive in human Phase I/II clinical trials. Compared to wild-type CHIK virus, (wt-CHIKV), both vaccines generated lower viral loads in a wide variety of tissues and organs, including the brain and leg muscle, but CHIKV/IRES exhibited marked restrictions in dissemination and viral loads compared to 181/clone25, and was never found outside the blood, spleen and muscle. Unlike wt-CHIKV, which caused disrupted splenic architecture and hepatic lesions, histopathological lesions were not observed in animals infected with either vaccine strain. To examine the stability of attenuation, both vaccines were passaged 5 times intracranially in infant A129 mice, then assessed for changes in virulence by comparing parental and passaged viruses for footpad swelling, weight stability and survival after subcutaneous infection. Whereas strain 181/clone25 p5 underwent a significant increase in virulence as measured by weight loss (from <10% to >30%) and mortality (from 0 to 100%), CHIKV/IRES underwent no detectible change in any measure of virulence (no significant weight loss and no mortality). These data indicate greater nonclinical safety of the CHIKV/IRES vaccine candidate compared to 181/clone25, further supporting its eligibility for human testing.

## Author Summary

Chikungunya fever is a reemerging, mosquito-borne viral disease that causes severe, debilitating, and often chronic arthralgia. The virus reemerged from Africa in 2004 and has since caused disease in millions of persons, including in over one million in the Americas since it arrived for the first time in modern scientific history in late 2013. An effective vaccine is critically needed to protect against this medically and economically devastating disease as well as to interrupt the human-mosquito transmission cycle. To further test a new, live-attenuated vaccine candidate for chikungunya fever, we conducted extensive preclinical safety evaluations using another vaccine candidate tested in humans, 181/clone 25, as a benchmark. The new vaccine candidate, CHIKV/IRES, replicated to lower levels in a mouse model and generated lesser signs of disease. Furthermore, it was more stably attenuated following mouse passages. These results support the further development of the new CHIKV/IRES vaccine candidate toward clinical testing in humans.

## Introduction

Chikungunya virus (CHIKV) is a reemerging arbovirus and the etiologic agent of chikungunya fever (CHIK). The virus belongs to the *Alphavirus* genus in the *Togaviridae* family. As an alphavirus, CHIKV particles are approximately 70 nm in diameter, and contain a single-stranded, positive-sense RNA genome of 11.8 kb [1]. The virus was discovered in Tanzania in 1952 by Robinson after responding to an isolated outbreak of febrile illness. This agent was later classified as a novel mosquito-borne virus that causes signs and symptoms similar to those associated with dengue fever [2–4]. The word chikungunya translates roughly from the African Kimakonde language to “that which bends up,” a reference to the hunched posture adopted by victims afflicted with severe arthralgia. CHIKF has a high attack rate, with only 2–25% of seropositive people remaining asymptomatic [5]. The symptoms and signs of CHIK include high fever, a maculopapular rash radiating outward from the trunk, intense arthralgia and myalgia, and in some rare cases neurological manifestations such as delirium and convulsions [5, 6].

Although it has emerged repeatedly from enzootic, mosquito-nonhuman primate (NHP) cycles in Sub-Saharan Africa for decades if not longer, CHIKV re-emerged with explosive outbreaks originating in Kenya in 2004 [7]. From this initial outbreak, CHIKV spread at an accelerated rate to make landfall in several islands of the Indian Ocean. La Reunión experienced a large outbreak, with approximately 38% of its population (300,000 people) contracting the illness [8]. In 2005, the Indian subcontinent began reporting multiple outbreaks of CHIKF and, shortly thereafter, Southeast Asia was also afflicted [9, 10]. Subsequently, also due to infected travelers, CHIKV was introduced into northern Italy and southern France, followed by autochthonous cases [11, 12]. In October of 2013, cases occurred for the first time in the Americas with autochthonous spread on the French portion of St. Martin Island [13, 14]. Subsequently, the Caribbean outbreak spread with local transmission on most Caribbean islands, northern South America, Central America, and Florida (http://www.cdc.gov/chikungunya/geo/americas.html)[15].

There is currently no licensed vaccine or treatment for CHIK. Patients generally receive only supportive care and non-steroidal anti-inflammatory drugs can alleviate some of the arthralgic pain and joint swelling. Vaccine development began in the 1960s with inactivated formulations generated from wild-type (wt) CHIKV strains [16–18], and later scientists at Walter Reed Army Institute of Research developed a live-attenuated vaccine, called 181/clone25, by serial passaging a Thai isolate through MRC-5 cells [19]. Although this vaccine was shown to be strongly immunogenic in small animal models and NHPs as well as in humans, Phase II clinical trials generated transient arthralgia in 5 of 59 participants [20], and virus isolated from the blood of vaccinees showed reversion of its attenuating mutations [21]. Later studies showed that strain 181/clone25 relies on only two point mutations for its attenuation phenotype, probably explaining its reactogenicity [21]. Subsequent vaccine development has included a wide variety of platforms [22], with a virus-like particle (VLP) formulation [23, 24] and a live-attenuated measles virus-vectored vaccine candidate [25, 26] proceeding through Phase I clinical trials with minimal side effects and complete seroconversion after two doses. However, the multiple dose requirement of these vaccines represents a limitation for the control of an explosively emerging viral disease and one that occurs mainly in resource-poor nations.

To develop a safe, rapidly immunogenic vaccine we capitalized on a strategy using the internal ribosome entry site (IRES) from encephalomyocarditis virus (EMCV) to attenuate alphaviruses [27]. The IRES is used to replace the subgenomic promoter for expression of the structural genes through internal initiation on the genomic RNA, greatly reducing structural protein production. Furthermore, the EMCV IRES functions inefficiently in insect cells, thereby preventing infection of mosquito vectors to enhance safety for use in non-endemic locations [28]. This attenuation strategy has been successfully implemented for CHIKV [29–32], Venezuelan [33, 34], and eastern equine encephalitis viruses [35] as well as the arthritogenic relative of CHIKV, Mayaro virus [36]. All of these vaccines have been demonstrated to be safe, immunogenic and efficacious in rodent models, and the CHIK version has been shown to be similarly effective in NHPs [37].

With the recent introduction and autochthonous CHIKV spread in the Americas there has been a resurgence of interest in developing a vaccine for CHIK. To this end, we expanded our studies with CHIKV/IRES to further test its preclinical safety using the A129 murine model developed by Courderc *et al*. [38]. A129 mice lack functional type I interferon receptors, rendering them susceptible to fatal disease following infection by wt CHIKV. We capitalized on this highly stringent model to examine levels of replication and tropism of the CHIKV/IRES vaccine candidate, and compared it to the highly immunogenic but mildly reactogenic 181/clone25 vaccine candidate [19, 20], as well as wt CHIKV. We also conducted an extended histopathological analysis not only to further assess CHIKV/IRES preclinical safety, but also to improve our understanding of CHIKV pathogenesis in this model. Finally, we conducted serial murine brain passages in neonatal A129 mice to assess the stability of genetic attenuation of CHIKV/IRES versus 181/clone25 attenuation.

## Methods

### Ethics Statement

The study was done in adherence to the Guide for the Care and Use of Laboratory Animals from the National Institutes of Health. All procedures were performed under protocol 02-09-068 approved by the Institutional Animal Care and Use Committee of the University of Texas Medical Branch.

### Cell Cultures and Viral Titrations

African green monkey kidney cells (Vero E6), were obtained from the American Type Cell Culture Collection, (Bethesda, MD.) The cells were maintained at 37°C with 5% CO_2_ in Dulbecco’s Modified Eagle’s Medium (DMEM) from Gibco (Grand Isle, NY). The medium was supplemented with penicillin/streptomycin from Gibco and 10% fetal bovine serum from Hyclone (Logan, UT). Viruses were titrated by plaque assay on these cells as previously described [39]. Briefly, the Vero cells were grown in to 6- or 12-well plates until they reached 95% confluency. The medium was then removed and 10-fold serial dilutions of virus were added to individual wells. After a one hour incubation at 37°C with 5% CO_2_, an overlay consisting of the medium listed above and 0.4% agarose was added. The plates were fixed 2–3 days later with formalin and were stained using 10% crystal violet.

### Virus Production/Acquisition

The CHIKV/IRES plasmid was produced previously as described by Plante *et al*. [29]. The wt-CHIKV/FfLuc plasmid was described previously [40]. Viruses were rescued by linearization of the plasmid and transcription of the viral genomic RNA using a Life Technology mMessage mMachine SP6 *in vitro* transcription kit (Waltham, MA). The resultant RNA was electroporated into Vero cells. Briefly, 2 confluent T-150 flasks Vero cells per electroporation were trypsinized and washed multiple times. The cells were then resuspended in 700 μl of PBS, approximately 4 μg of RNA was added and transferred to a 4 mm cuvette from Molecular Bioproducts (San Diego, CA). The RNA/Vero cell suspension was electroporated using 3 pulses at 250 V, 10ms, one-second intervals in a BTX830 electroporator from Harvard Apparatus (Holliston, MA). Electroporated cells were then placed into a T-75 flask and virus was harvested 30–36 hours later. The vaccine virus derived from electroporated cells was used without further passage.

### Animal Studies

The A129 type I interferon receptor^-/-^ mice were kindly provided by Slobodan Paessler at the University of Texas Medical Branch. The mice were bred in triads (2 female 1 male) and were weaned at 21 days post-birth. Adult 10-week-old animals were used for pathogenesis and in vivo imaging system (IVIS) experiments, whereas neonatal 2-day-old animals were used for serial brain passages. The lactating mothers were left in the cage during any procedure that required neonatal mice.

Histopathologic studies used adult 10-week-old A129 mice that received a 10^4^ PFU total dose delivered intradermally (ID) into the left footpad in a 10 μl volume. Footpads were measured daily for 14 days and animals were sacrificed on days 1–4, 8, 14, 21, or 28 post-infection after anesthetization with isoflurane. For a single experiment, infected cohorts included 3 animals per virus group for the day 1, 2, 3, 7, 14, 21, and 28 post-infection harvests. Day 4 harvests had 4 animals per infected cohort. A single mock-infected mouse was included at each timepoint. Blood was collected by cutting the aortic arch then transcardially perfusing mice with ~40 ml of PBS at a flow rate of 2 ml/min. The major organs were then harvested and a sample of the muscle from the left hamstring was extracted. Half each sample was placed in 10% formalin for histopathological analysis, while the remaining half (same organ or contralateral) was placed in DMEM containing 5% FBS for viral load assays.

An IVIS experiment was completed using 10-week-old A129 animals to visualize patterns of CHIKV replication in vaccinated mice. Each cohort included four animals except for the mock vaccinated, wt-CHIKV cohort, which included two animals. At 26 days post-vaccination, the mice were prepared by shaving the dorsal and ventral surfaces; a small strip of hair was left on the dorsal surface adjacent to the front limbs, and extra bedding was supplied for comfort. At day 30 post-vaccination, the mice were injected ID in the footpad as described above with either wt-CHIKV or wt-CHIKV/FfLuc. Weights and footpad measurements were collected daily post-challenge for the duration of the study. On days 1–4 post-challenge, 170 μl of luciferin substrate (GoldBio, St. Louis, MO) at a concentration of 15mg/ml were injected into each animal IP. The animals were then returned to their cages for 7 minutes before anesthetization with inhalational isoflurane and imaging using the Xenogen IVIS system (Lincolnshire, United Kingdom) and LivingImage software from Perkin Elmer (Waltham, MA). At day 3 post-challenge some animals were sacrificed and their tissues were harvested for analysis as described previously. Results represent a single experiment.

To assess the stability of attenuation, the CHIKV/IRES and 181/clone25 vaccine strains were passaged intracranially (IC) in 2-3-day-old mice. Each vaccine strain was passaged in 2 independent experimental series. Mouse litters were randomized at 2 days of age, then cohorts of 3 mice were injected IC with 10 μl containing 10^4^ PFU. The animals were then sacrificed 30–36 hr later and their brains were homogenized and titrated. The highest titer of the three possible samples was chosen for each experiment to continue the IC passage series after clarification through centrifugation dilution to a titer of 10^6^ PFU/ml. After 5 brain passages, passaged vaccine strains was tested for changes in virulence by comparing mortality rates with the parental strain. Each passaged virus and its parent was tested in 3–4 animals, alongside 5 wt-CHIKV and 2 PBS controls.

### Histopathological Analysis

The procedure for fixation and staining was described previously [29]. Briefly, tissues were fixed in 10% buffered formalin (RICCA, Arlington TX) for a minimum of 72 hours. Samples that contained bone, such as whole leg, were then decalcified overnight (VWR, Radnar, PA). The tissues were then embedded in paraffin wax and 5μm sections were prepared. The samples were hydrated using an ethanol gradient and stained with hematoxylin and eosin.

### Sequence Analysis

The vaccine strains were sequenced following serial brain passages. Consensus sequences was determined by directly sequencing reverse transcription-polymerase chain reaction (RT-PCR; conditions and primer sequences available from the authors upon request) amplicons generated from RNA extracted from brain homogenates using TRIzol LS (Life Technologies) and the Titan One Tube RT-PCR System kit from Roche (Penzberg, Germany). Sequencing reactions were performed using the BigDye kit from Applied Biosystems (Foster City, CA) and amplicon DNA was purified using EdgeBio Sequence Cleanup columns (Gaithersburg, MD) and sequenced on an Applied Biosystems 3500 Genetic Analyzer. Vero cell plaque purified virus was also collected for sequencing.

## Results

### Vaccine Candidate Tropism and Signs of Infection

**Fig 1:**
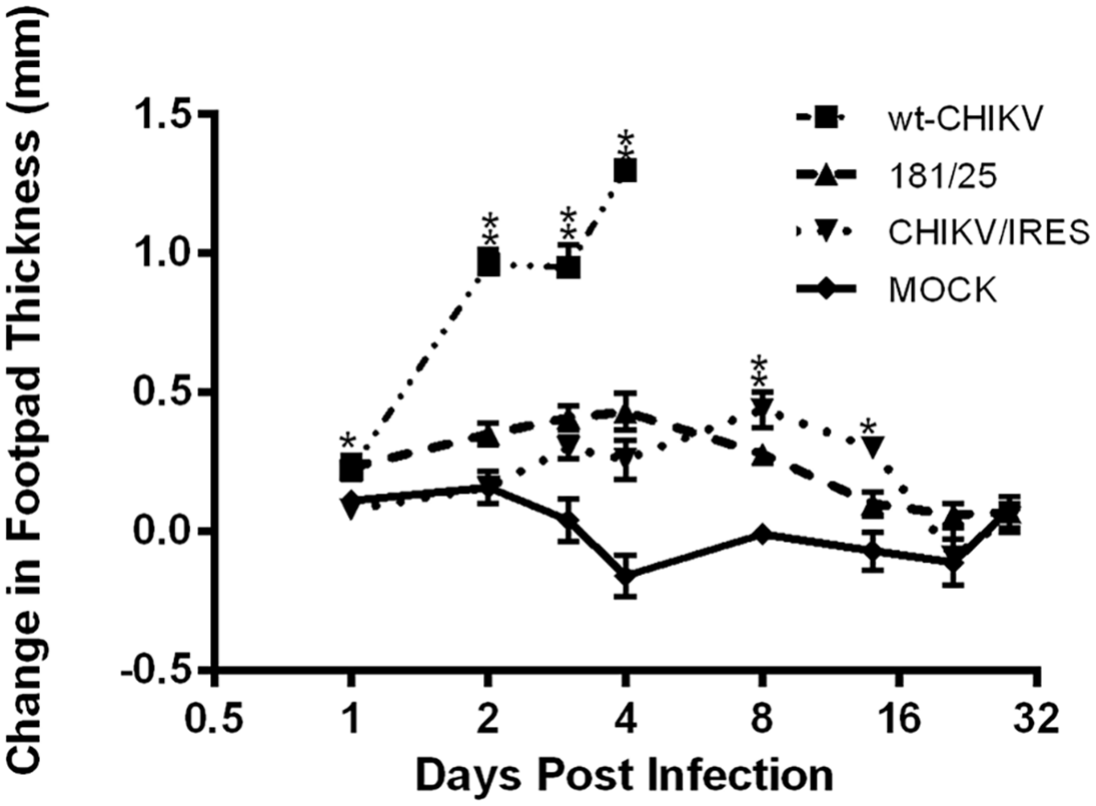
Swelling of the ipsilateral footpads of A129 mice after infection with 10^4^ PFU of wild-type CHIKV, or the 181/clone25 or CHIKV/IRES vaccine strains delivered in the footpad. Statistical analysis was performed with one-way ANOVA. Single asterisks indicate p<0.05. Double asterisks indicate p<0.001.

To assess the tropism of wt-CHIKV and the 181/clone25 and CHIKV/IRES vaccines, a serial sacrifice study was completed using the 10-week-old A129 mouse model inoculated in the footpad with 10^4^ PFU. Ipsilateral footpad swelling was measured and tissues were harvested from 3 animals on days 1–4, 8, 14, 21, and 28 post-inoculation to determine viral loads by plaque assay. The first 4 days were used to compare the vaccines’ tropisms and histopathological findings versus wt-CHIKV. The later time points, at which the wt-CHIKV-infected animals had already succumbed to illness, were used to compare the two vaccine strains. By day 1 after infection, minor footpad swelling was observed in both vaccine cohorts (Fig 1). This phenotype has been observed previously [29] and was attributed to mechanical disruption during the footpad inoculation. The day 2 measurements began to show a difference between the wt-CHIKV-infected animals and the vaccine cohorts, with moderate to severe swelling maintained in the former through day 4. During the later time points, the vaccinated animals maintained mild swelling through the 14-day timepoint. By the 21-day timepoint no residual swelling was observed in either vaccine cohort.

**Fig 2:**
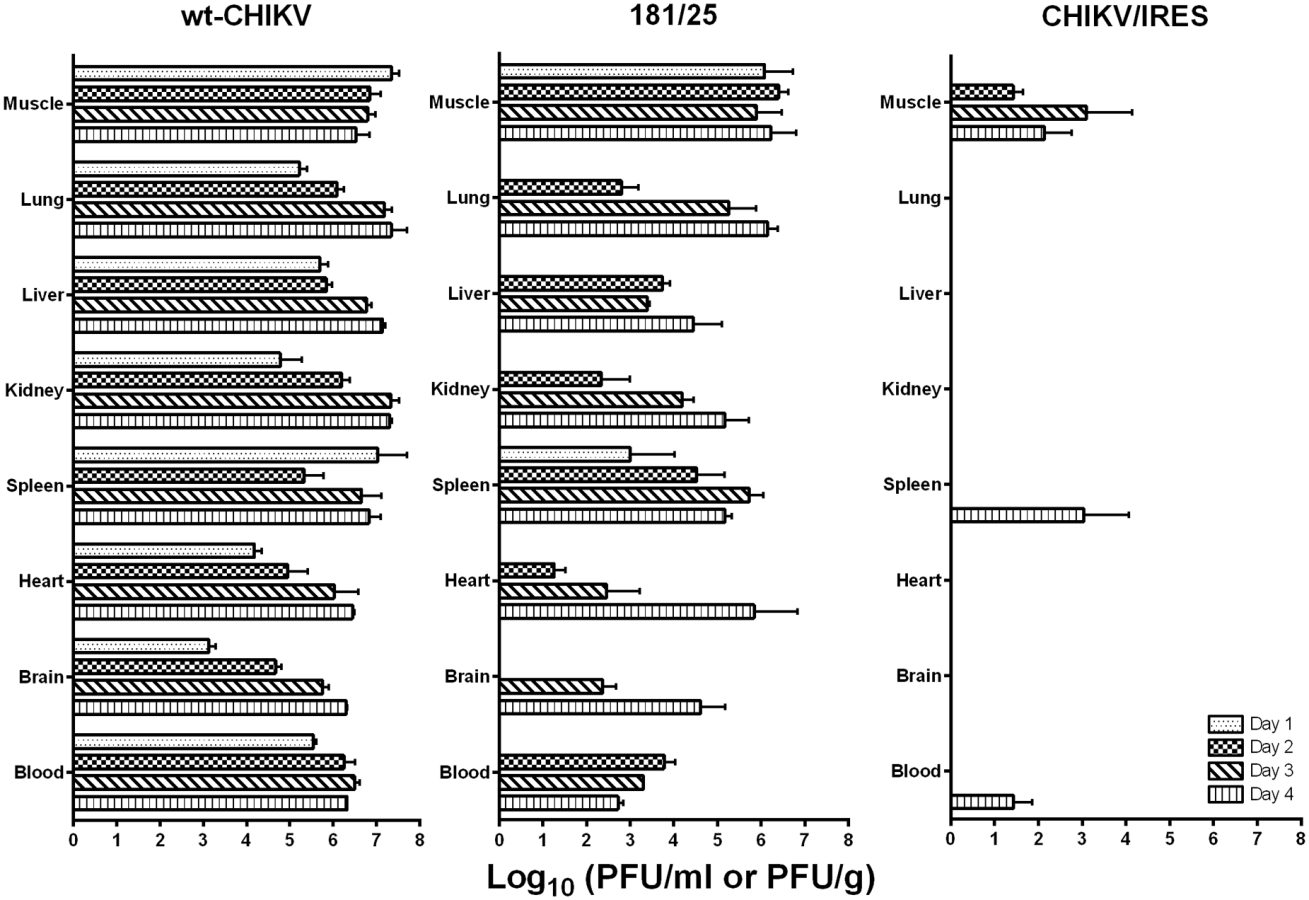
Mean viral loads in A129 mice after infection with 10^4^ PFU of wild-type CHIKV, or the 181/clone25 or CHIKV/IRES vaccine candidate strains delivered in the footpad. The limit of detection of the plaque assays was 60 PFU/g or ml. Data were analyzed by one-way ANOVA with a Bonferroni post hoc test for pairwise comparisons.

Mice were sacrificed for terminal bleeds, and transcardial perfusions were performed, followed by necropsies to collect the major organs for titration and histopathological analyses. Virus was detected in some animals of all cohorts up to the day 4 timepoint (Fig 2). The two vaccine groups had tissues collected from later time points, but by day 8 there was no detectable virus present by plaque assay in any of the tested tissues. The wt-CHIKV-infected animals produced a systemic infection by day one. The highest titers were found in the hamstring muscle and spleen of these animals. The brain had the lowest viral load on day one, approximately 3 log_10_ PFU/g. The viral loads in the wt-CHIKV-infected animals increased through day 4 until the tissues uniformly contained large viral loads of approximately 6-7log_10_ PFU/g (tissue) or PFU/ml (serum).

The 181/clone25 vaccine strain also replicated well in the A129 model (Fig 2). By day 1 post infection animals had detectable viral loads in the muscle and spleen (Fig 2). The hamstring had the higher viral load, which was 6 log_10_ PFU/g compared the spleens 3 log_10_ PFU/g. By day 2 the animals had a high levels of viremia and all tissues except the brain contained virus. This trend continued by day 3, when the animals were found to have a systemic infection with brain samples now containing virus. Overall virus production continued to increase through day 4, with the highest titer tissues approaching 7 log_10_ PFU/g. However, 181/clone25 virus was cleared to undetectable levels in all tissues examined by day 8. Day 14, 21 and 28 samples were also negative by plaque titration (<100 PFU/g).

In contrast to wt-CHIKV and 181/clone25, the CHIKV/IRES-infected animals never developed high virus loads (Fig 2). CHIKV/IRES could not be detected in any of the tissues tested on day 1. By day 2, 2/3 animals had low viral loads detected in the muscle, slightly above our limit of detection. By day 3, virus could still only be detected in the muscle, with the titer increased to 3 log_10_ PFU/g. Day 4 samples revealed slow spread to the spleen (2/3 animals) and one animal had a slight viremia. CHIKV/IRES was not detected at later timepoints. One-way ANOVA indicated a significant difference among the 3 virus/vaccine cohorts for the timepoints and tissues tested. Using Bonferroni post hoc analyses for pair-wise comparisons, virus titers in all tissues except for muscle were statistically indistinguishable between the vaccine strains on day 1. In the muscle, the 181/25 clone was statistically indistinguishable from wt-CHIKV, and CHIKV/IRES was statistically different from both the 181/25 clone and wt-CHIKV. The CHIKV/IRES cohort titers remained significantly lower than wt-CHIKV at all timepoints in all tissues. The day 2 and 3 181/clone 25 animals had similar viral loads in the muscle and spleen compared to wt-CHIKV. The trend continued by day 4 the two viruses now present at similar titers in the brain, heart, spleen and muscle.

**Fig 3:**
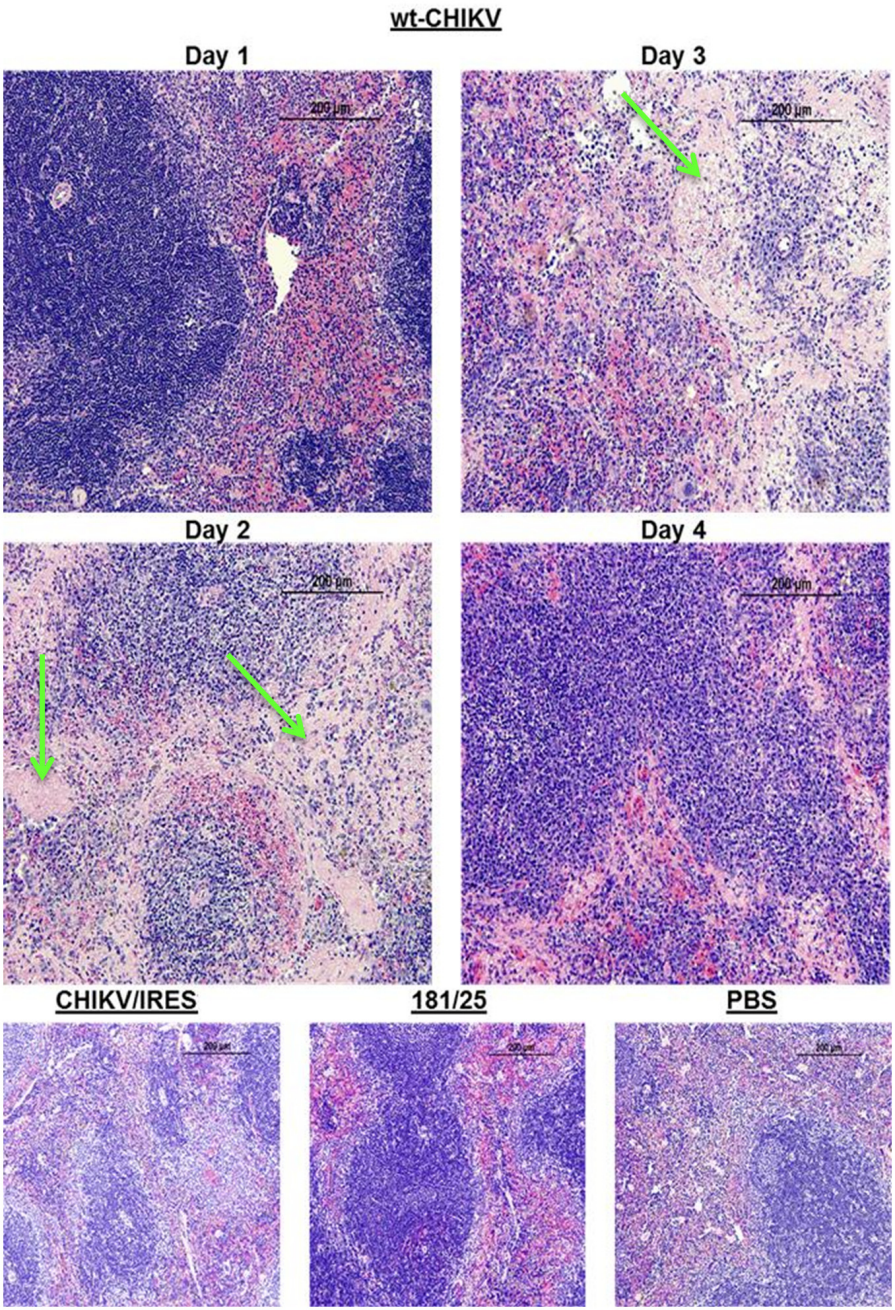
Representative histopathologic spleen lesions in A129 mice after infection with 10^4^ PFU of wild-type CHIKV, or the 181/clone25 or CHIKV/IRES vaccine candidate strains delivered in the footpad. Images for CHIKV/IRES, 181/clone25 and PBS are from 3 days post-infection. Green arrows indicate the deposition of proteinacious debris.

**Fig 4:**
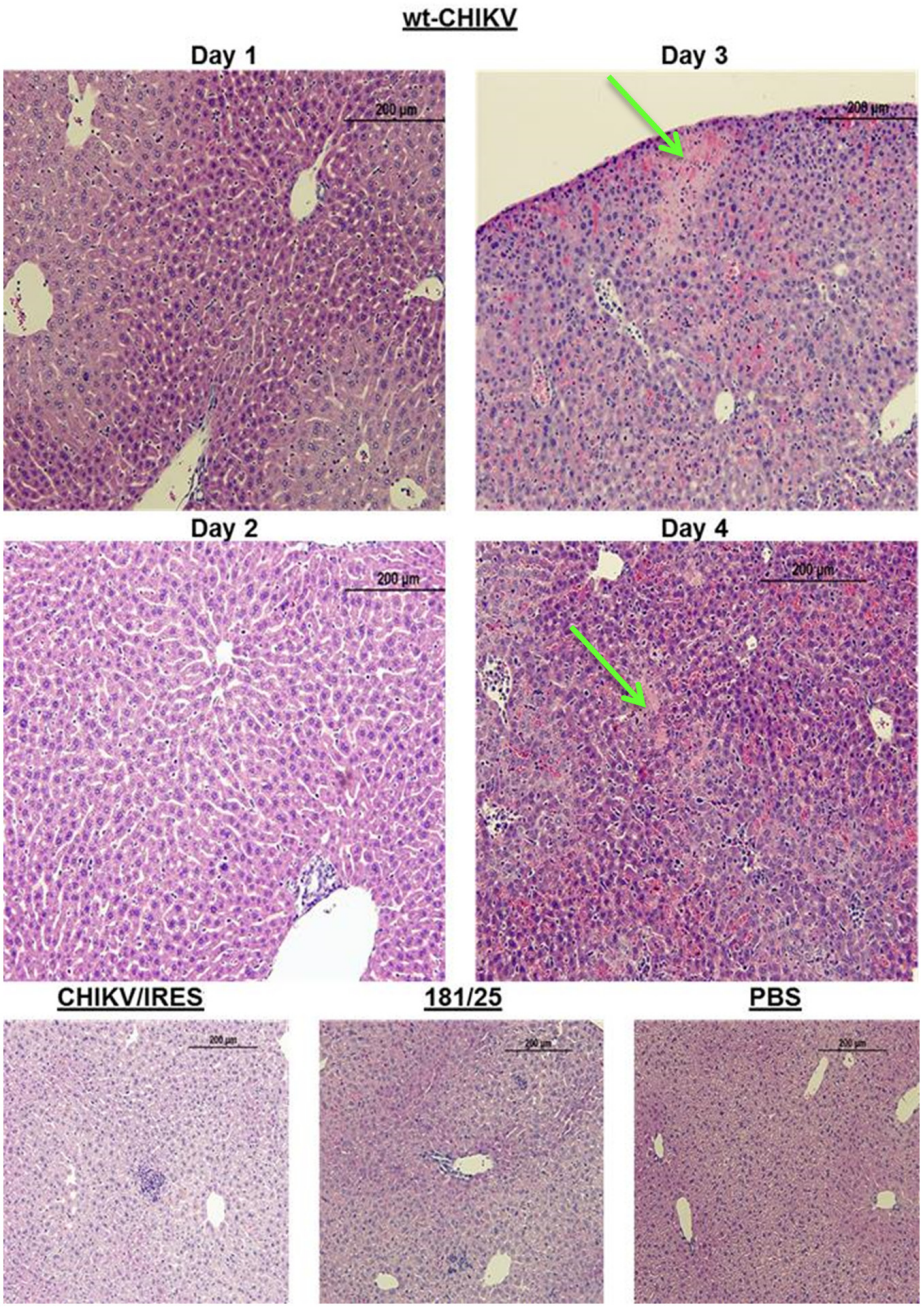
Representative histopathologic liver lesions in A129 mice after infection with 10^4^ PFU of wild-type CHIKV, or the 181/clone25 or CHIKV/IRES vaccine candidate strains delivered in the footpad. Images for CHIKV/IRES, 181/clone25 and PBS are from 3 days post-infection. Green arrows indicate hepatic lesions.

**Fig 5:**
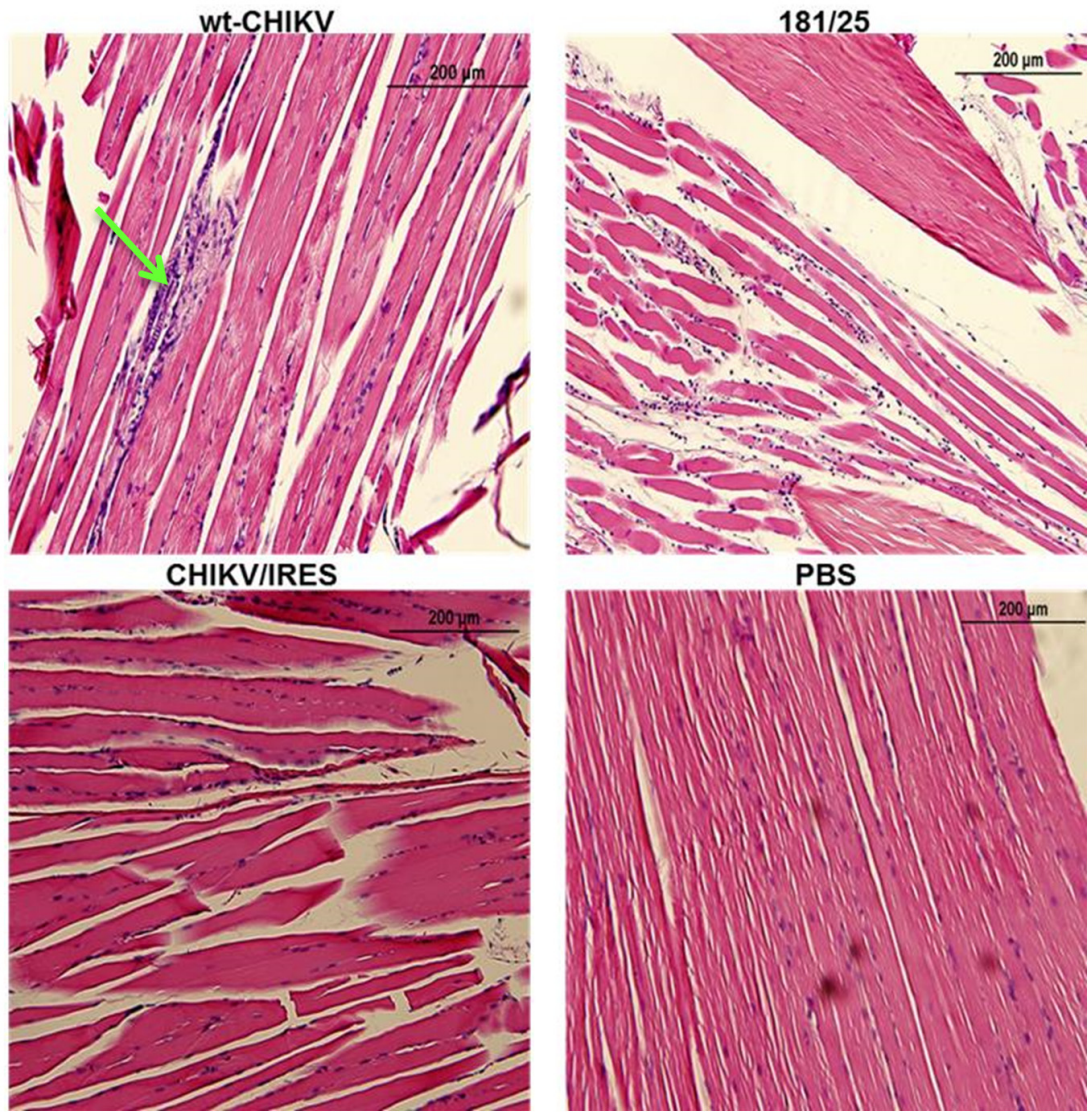
Representative leg muscle histopathologic lesions in A129 mice 3 days after infection with 10^4^ PFU of wild-type CHIKV, or the 181/clone25 or CHIKV/IRES vaccine candidate strains delivered in the footpad. Green arrows indicate sites of focal myositis.

**Fig 6:**
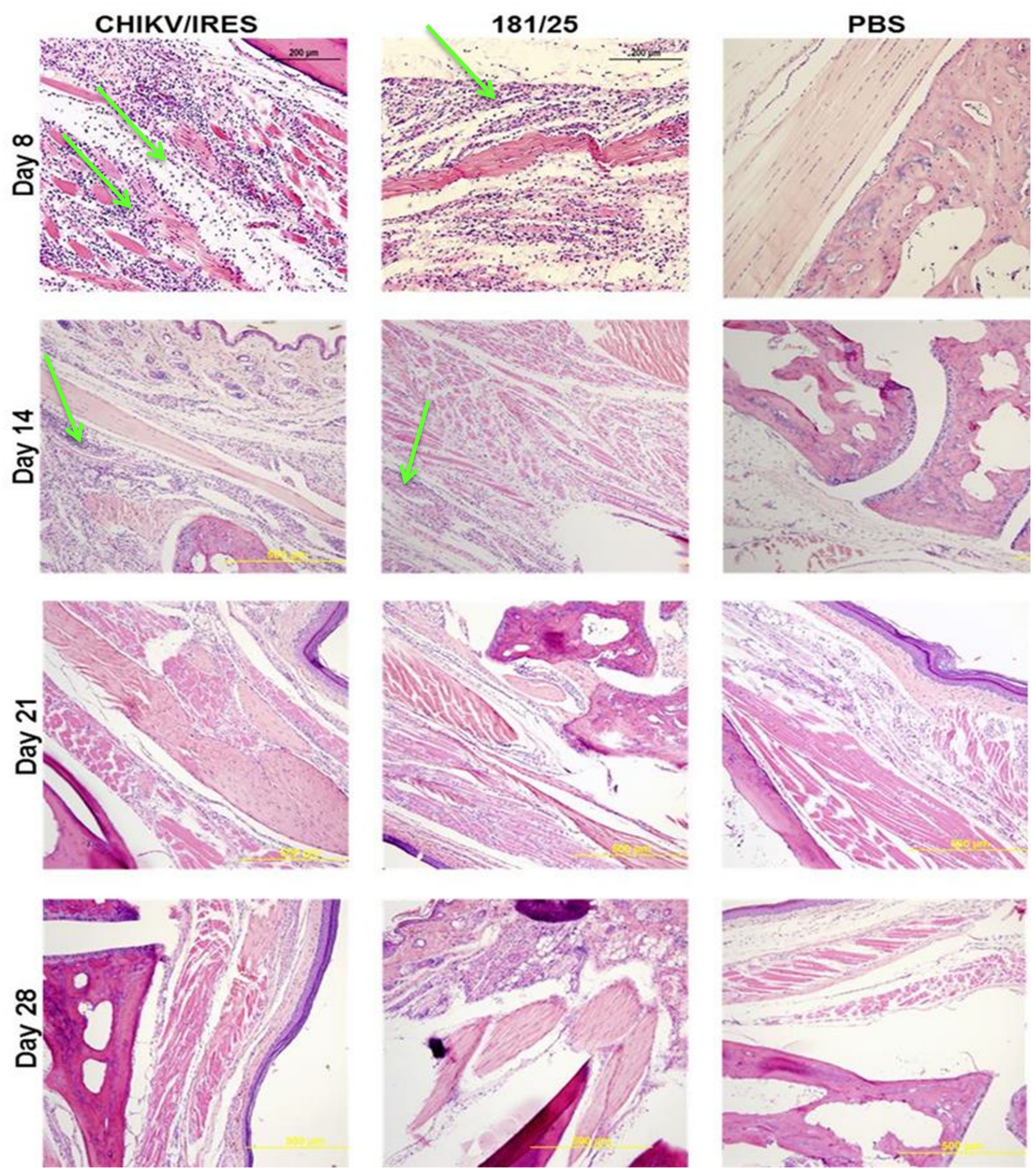
Representative leg muscle histopathologic lesions in A129 mice 14 days after infection with 10^4^ PFU of the 181/clone25 or CHIKV/IRES vaccine candidate strains or PBS delivered in the footpad. Green arrows indicate cellular infiltrate and moderate myositis.

Histopathological analyses were completed on all major organs at each time point. Only the spleen, liver, and leg muscle showed remarkable lesions. None of the animals that received either vaccine or PBS exhibited lesions in the spleen during the course of the study. However, the animals that received wt-CHIKV exhibited disrupted splenic architecture with depletion of the white pulp and deposition of proteinaceous debris by day 2, which persisted through day 3 (Fig 3). By day 4 the spleen began to recover histologically (Fig 3). The wt-CHIKV-infected animals also showed small hepatic lesions, which appeared on day 3 and persisted at least until day 4 (Fig 4). Neither vaccine cohort showed any hepatic lesions during the study, similar to the PBS cohort. Likewise, the histological findings for the leg after 181/clone25, CHIKV/IRES, or PBS infections were unremarkable through day 4 (Fig 5). Mild foci of myositis could be seen in the calf muscles of the wt-CHIKV-infected mice at day 4; the later timepoints revealed the development of moderate myositis and cellulitis in animals infected with each vaccine strain on day 8 and to a lesser degree on day 14 (Fig 6). These animals showed no outward signs of disease during this period (none with hunched posture, ruffled fur, or lethargy). This inflammation was generally cleared by day 21 and no pathologic findings could be detected by day 28.

### Virus Stability following Serial Mouse Brain Passages

**Fig 7:**
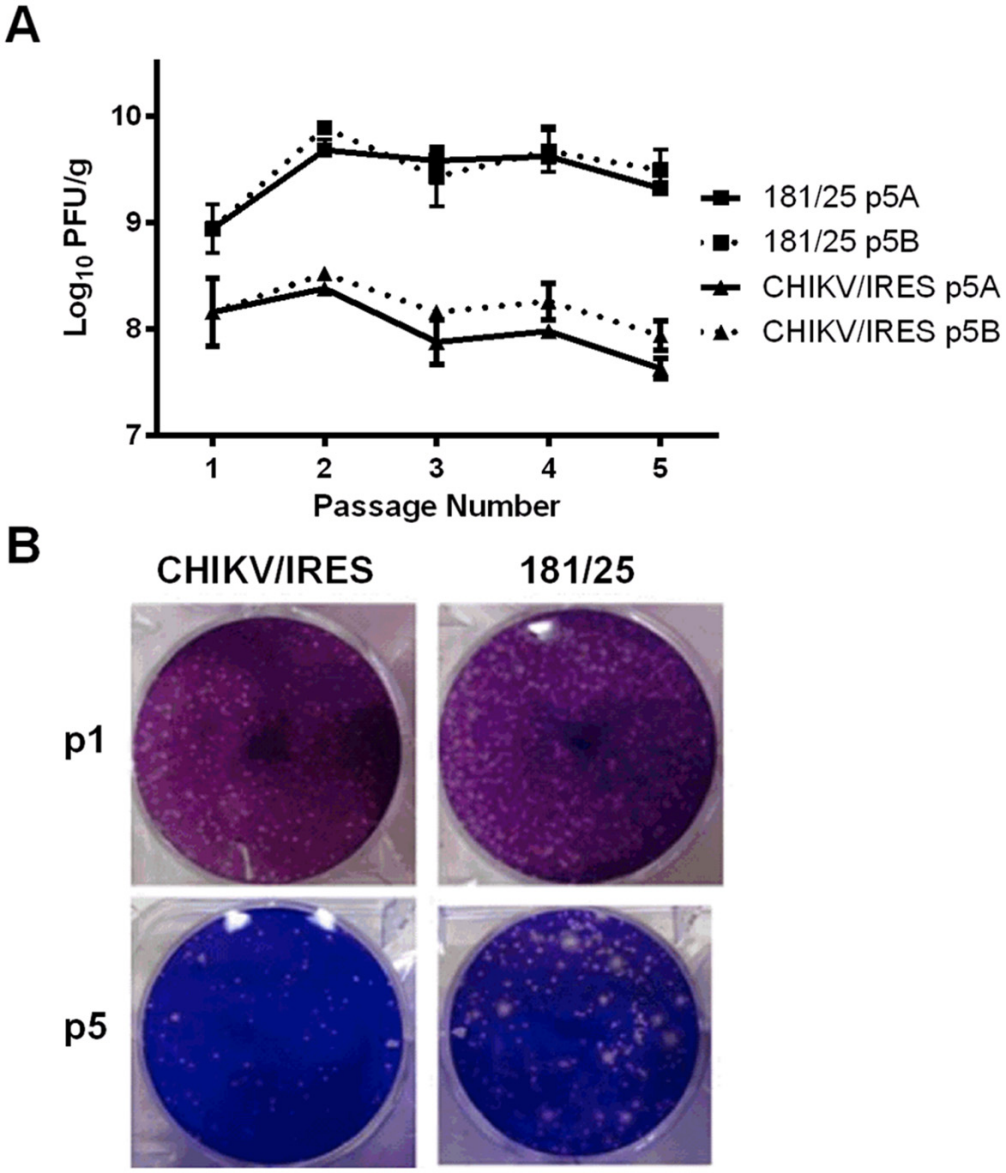
A. Titers of CHIKV vaccine candidate strains in the brains of 2-day-old A129 mice after serial passages. B. Representative plaque morphology of mouse-passaged CHIKV vaccine strains. Data was analyzed by one-way ANOVA with a Bonferroni post hoc test for pairwise comparisons.

To assess the genetic stability of the CHIKV/IRES and 181/clone25 candidate vaccines, two independent passage series of each were performed in neonatal A129 mice inoculated intracranially. Peripheral inoculation did not generate enough replication to allow for serial passages, and strain CHIKV/IRES did not replicate to sufficient titers in the brains of wt mice to permit serial propagation. The viruses were inoculated intracranially into the brains of 2-day-old A129 mice in a volume of 10 μl containing 10^4^ PFU, then the animals were euthanized and brain tissue was harvested 36 hours later. Each cohort consisted of 2 pups and their lactating mother. After titration, the animal with the highest viral brain titer was chosen to continue the passage series after the appropriate dilutions to inoculate 10^4^ pfu into each of the next pair of mice. Overall, brain titers for the 181/clone25 vaccine strain were ca. 10-100-fold higher than those of CHIKV/IRES (Fig 7A). There was significant variation among the groups in all passages by one-way ANOVA. However, with the exception of the day 2 samples for 181/clone25, there was no significant variation between independent passages of each virus strain by Bonferroni post-hoc analysis. Plaque morphology was also determined throughout these passages, and some noticeably larger plaques appeared as early as the second passage for the 181/clone25 vaccine. In contrast, plaques of the CHIKV/IRES vaccine candidate remained relatively constant in size throughout the passages (Fig 7B).

Following 5 serial passages, viruses were analyzed for changes in virulence in a 6-7-week-old A129 mouse model inoculated intradermally in the left rear footpad (intracranial inoculation of strain 181/clone25 resulted in rapid death with little opportunity to observe changes in survival time following passages) using 10^4^ PFU. Parent virus strains derived from electroporated stocks of each vaccine were compared to the 5^th^ mouse passages, as well as to PBS controls. The animals were observed for 13 days post infection and weight change, footpad swelling, and mortality were noted.

**Fig 8:**
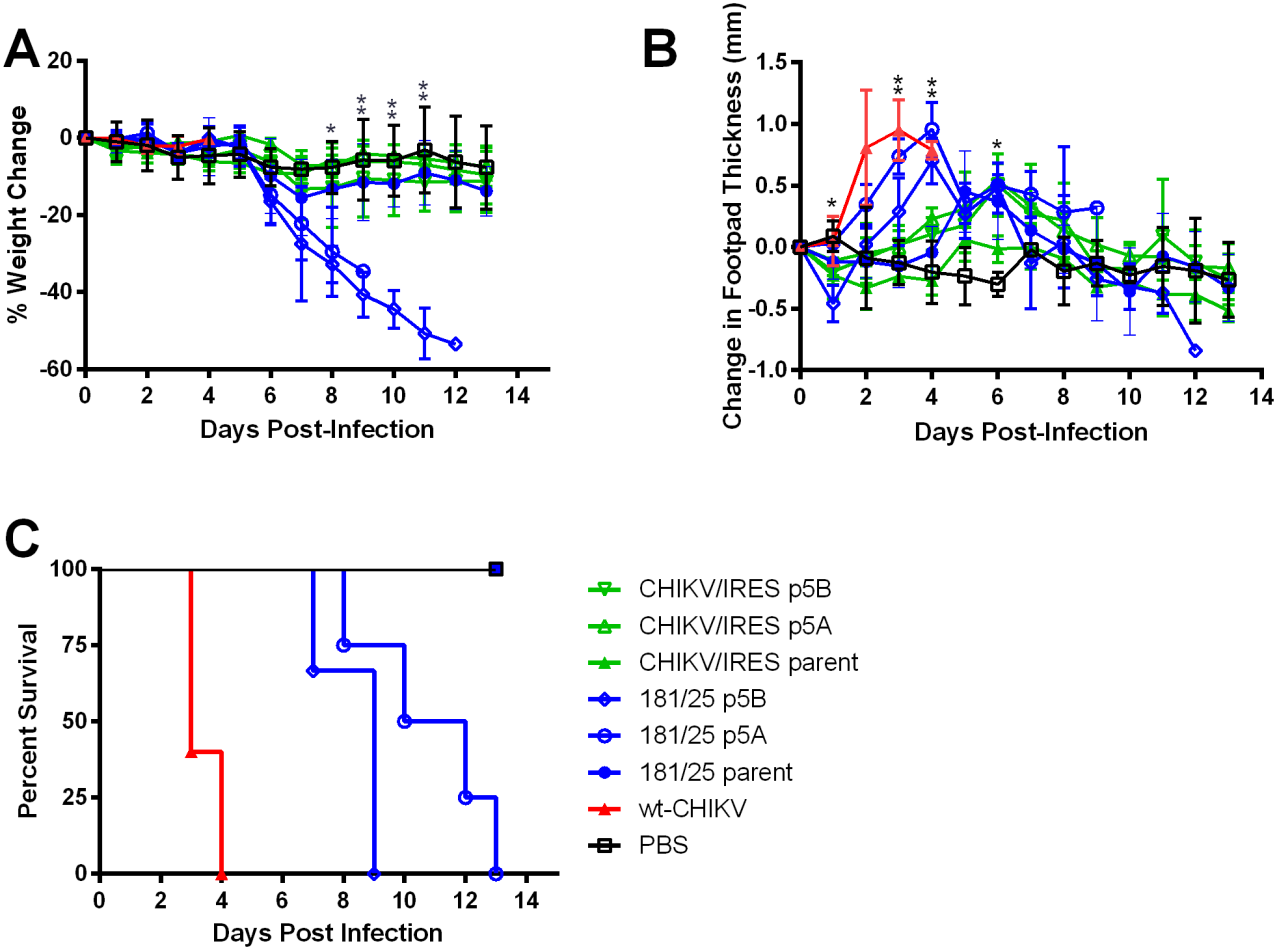
A. Weight change, B. Footpad swelling, and; C. Survival of 6-7-week-old A129 mice inoculated intradermally in the footpad with 10^4^ PFU of wt CHIKV, parent vaccine strains, or 5^th^ intracranial passage vaccine strains from in duplicate, serial passage series (A and B). Statistical analysis for panels A and B was performed with one-way ANOVA. Single asterisks indicate p<0.05. Double asterisks indicate p<0.001. Statistical analysis for panel 8C was completed by Kaplan Meier.

When weights of mice infected with the passaged viruses were compared, mice that received p5 of 181/clone25 began to lose weight at day 6, and continuing through the end of the study (Fig 8A). In contrast, the p5 CHIKV/IRES-infected mice did not differ significantly in weight change compared to the PBS-injected controls or the unpassaged vaccine strains. The two mouse-passaged lines (p5A, p5B) of the CHIKV/IRES vaccine candidate caused similar levels of footpad swelling compared to the parent virus and to the PBS control, with significantly less swelling compared PBS only on day 6 (Fig 8B). On days 1, 3, and 4, wt-CHIKV produced more swelling that either vaccine or PBS. Unlike CHIKV/IRES, both passaged181/clone25 strains exhibited an altered footpad swelling phenotype, with the latter causing increased swelling similar to that induced by wt-CHIKV. There were also significant differences in mortality among the 8 treatments as calculated by a Kaplan Meier test. Neither of the parent vaccines or either p5 replicate of the CHIKV/IRES vaccine candidate, or the PBS control caused any mortality (Fig 8). In contrast, both p5 181/clone25 replicates became 100% lethal. Interestingly, the p5B 181/clone25 strain appeared to be more virulent, killing all the animals tested by day 9 whereas some p5A-infected animals survived 13 days. However, the Kaplan Meier test showed a p-value of only 0.11 for this difference in average survival. Because stochastic events are likely involved in reversion or pseudoreversion to virulence, it would not be surprising to see different trajectories in the virulence increases when only 2 attenuating point mutations [21] must be overcome to regain virulence in strain 181/clone25.

To investigate the mechanisms of reversion to virulence or stability, we consensus sequenced the complete genomic RNA extracted from the 5^th^ mouse passage of each series (2 parallel series for 181/clone 25 and CHIKV/IRES) using RT-PCR followed by Sanger amplicon sequencing. The passaged CHIKV/IRES viruses had no consensus mutations located in the open reading frames. In contrast, the passaged 181/clone25 vaccine viruses acquired four mutations in the nonstructural protein genes, two encoding amino acid substitutions in the nsP1, all found in both parallel passage series, and generally in mixed populations (Table 1).

**Table 1:** Consensus substitutions acquired by the 181/clone25 vaccine strain during serial mouse passages.

Passage series	Genomic nucleotide position	Mutation	Mixed Population?	Gene and codon position	Amino Acid substitution	Plaque size
5A, 5B	978	T→C	Yes	nsP1-301	Ile→Thr	N/A
5A, 5B	1016	A→T	Yes	nsP1-314	Met→Leu	N/A
5A, 5B	3706	G→A	No	nsP2-675	synonymous	N/A
5A, 5B	6043	T→C	Yes	nsP4-126	synonymous	N/A
5A	8780	G→C	No	E2-80	Arg→Thr	Large
5A	8280	A→G	No	Capsid-238	synonymous	Small
5B	8712	G→T	No	E2-57	Lys→Asn	Large
5B	8785	A→G	No	E2-82	Arg→Gly	Large
5B	8564	T→C	No	E2-8	Val→Ala	Small

Because the attenuating mutations of vaccine strain 181/clone25 are found in the E2 envelope glycoprotein [21], we harvested virus from 2 large and 2 small plaques for each p5 virus. For each of the 4 plaque-purified stocks, the PE2 gene region, spanning from the 3’ end of capsid gene to the 5’ end of 6K, was sequenced using the Sanger method [21]. The plaque purified CHIKV/IRES sequences derived from the unpassaged parent strain and each p5 derivative were unchanged compared to that of the parent clone. However, the 181/clone25 mouse p5 viruses acquired multiple E2 gene mutations (Table 1). In each case, the 2 sequences derived from the large plaques sampled from a given p5 brain harvest contained the same E2 mutation; the same was true for the small plaques. The p5A of 181/clone25 virus had an E2 substitution of R80T, involving a reduction in change near residue 82 where the vaccine strain had acquired a G82R substitution that is its major attenuation determinant [21]. In 181/clone25 p5B, there was direct reversion of amino acid 82 to Gly, and a second loss of charge mutation at E2-K57N. The substitutions resulting in the loss of positive charge suggest that increased mouse virulence was mediated by a reduction in heparan sulfate binding, as has been shown to differ between 181/clone25 and wt-CHIKV [41, 42]. When these mutations were mapped using Pymol on the E2 glycoprotein, all three occurred on the apical side of the protein in domain B [43](S1 Fig), consistent in their putative role in binding to cellular receptors [43].

### In-Vitro Imaging to Evaluate Immune Protection

**Fig 9:**
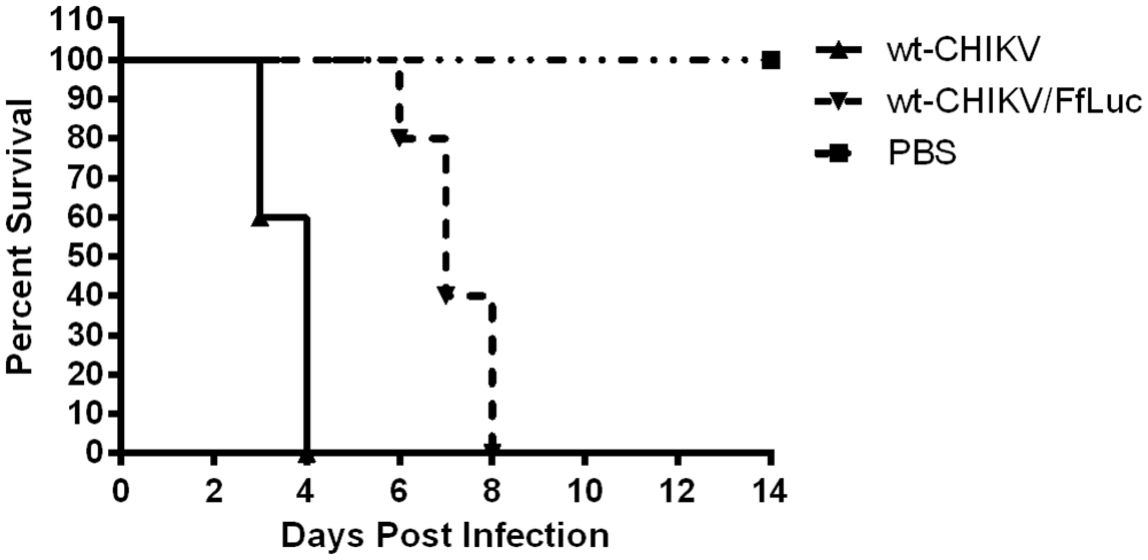
Mortality in 10-week-old A129 mice after infection in the footpad with 10^4^ PFU of wt-CHIKV or wt-CHIKV/FfLuc. Statistical analysis was performed by Kaplan Meier.

The highly efficacious nature of the CHIKV/IRES vaccine candidate has been previously demonstrated in mice and nonhuman primates [29, 37]. However, none of these studies evaluated exhaustively the replication of challenge CHIKV in vaccinated animals. In an attempt to evaluate the fate of challenge virus without sacrificing large numbers of animals, we used a wt-CHIKV strain that expresses the firefly luciferase gene fused directly to the capsid protein [40]. This virus was initially compared to our standard, wt-CHIKV in a mortality study to determine the effect of the reporter gene on virulence using 10-week-old A129 mice infected with a 10^4^ pfu dose in the footpad. Both viruses were 100% lethal; however wt-CHIKV/FfLuc exhibited a statistically significant (Kaplan Meier) delay in the mean time to death (ca. 6 versus 3 days) compared wt-CHIKV (Fig 9).

Because both viruses caused 100% mortality, we used CHIKV/FfLuc virus for challenge of mice 30 days after CHIKV/IRES or PBS vaccination. One cohort was challenged with wt-CHIKV to determine if autofluorescence would confound imaging. The mice were given the fluorescein substrate daily and observed using the IVIS system. When the whole animal was imaged, a strong luminescence signal was present in the inoculated footpad of the PBS-vaccinated animals that were challenged CHIKV/FfLuc virus, but as expected no signal was detected in the animals that were challenged with wt-CHIKV (S2 Fig). The animals that received the CHIKV/IRES vaccine candidate and were then challenged with CHIKV/FfLuc also produced no detectable signal.

The footpad signal from the PBS-vaccinated, CHIIKV/FfLuc-challenged animals was so high that it masked weaker signals from elsewhere in the body (S3 Fig). When the animals’ inoculated footpads were masked, a strong signal was detected in the musculature and splenic region on days 1–4. In contrast, the CHIKV/IRES vaccine candidate completely protected against CHIKV/FfLuc replication detected by luciferase activity. The PBS-vaccinated animals did have lower levels of infectious virus replication following CHIKV/FfLuc challenge, compared to the wt-CHIKV (S4 Fig). However, in the absence of vaccination, the infection was systemic by day 3 and caused minor splenic disruption (S5 Fig).

## Discussion

The ideal vaccine for an explosively emerging viral disease like CHIK will cause no detectable disease after administration, will generate rapid and durable immunity after a single dose, and will prevent or greatly reduce the replication of challenge infections to reduce transmission (since humans are the only amplification hosts in the urban cycle). The vaccine will also remain stably attenuated during use in large numbers of vaccinees, especially for viruses like CHIKV that place tens-of-millions of persons at natural risk for severe and chronic arthralgia. A live-attenuated Chikungunya vaccine may be capable of meeting these goals [22].

We previously described a new, live-attenuated vaccine candidate, CHIKV/IRES, developed by inactivating the subgenomic promoter and expressing the CHIKV structural proteins from the genomic RNA using IRES-mediated translation. This vaccine was shown to be well attenuated, immunogenic and efficacious in protecting against CHIK virus (CHIKV) challenge of mice [29] and nonhuman primates [37]. In this study, we performed more extensive studies to further evaluate CHIKV/IRES preclinical efficacy and safety using the A129 interferon-α/β receptor deficient murine model in which wt-CHIK produces a lethal infection. We also used the 181/clone25 live-attenuated vaccine strain as a benchmark, which exhibited excellent immunogenicity but some reactogenicity in Phase II human trials [20], presumably due to the presence of only 2 attenuating point mutations [21].

To evaluate preclinical safety, we performed serial sacrifice experiments with adult A129 mice to measure viral loads in a wide variety of tissues and organs 1–8 days after vaccination. The CHIKV/IRES vaccine candidate replicated to far lower levels than wt-CHIKV, which generated a rapid and systemic infection by day one. The CHIKV/IRES viral loads were also generally much lower than those of 181/clone25, which were intermediate between this vaccine and wt-CHIKV. Footpad swelling, another measure of virulence, was also lower following inoculation of both vaccines compared to wt-CHIKV.

To assess the stability of CHIKV/IRES attenuation, we attempted to enrich for reversion to virulence by performing serial brain passages in 2-day-old A129 mice. Following 2 independent experiments involving 5 serial passages, no increase in virulence, as measured by mortality, weight loss or footpad swelling, was detected in the CHIKV/IRES vaccine candidate strain (Fig 8). In contrast, strain 181/clone25 consistently became 100% lethal and increased its ability to cause weight loss and footpad swelling. This presumed mechanism of strain 181/clone25 instability, reversions or pseudoreversions in the attenuating E2 envelope glycoprotein substitutions that accompanied its development during serial MRC5 cell passages, were confirmed by the identification of loss-of-charge substitutions of the same or nearby E2 residues during serial mouse passages. In contrast, no consensus mutations occurred in the open reading frames of either of the CHIKV/IRES mouse passage series. The only mutation detected was in the length of a polyA tract within the IRES itself, which is not translated.

Finally, to assess the ability of the candidate CHIKV/IRES vaccine to inhibit challenge virus replication, a wt-CHIKV construct was modified to fuse the luciferase gene to the CHIKV capsid, producing a virus that expressed luciferase during replication in mice. Using this system and IVIS, we demonstrated that immunization by the candidate CHIKV/IRES vaccine protected completely against detectable luciferase expression, suggesting nearly sterilizing immunity to challenge with the CHIKV/Ffluc virus. This is an important finding because, in addition to preventing disease, a CHIK vaccine should diminish CHIKV viremia after exposure of vaccinated subjects to interrupt the urban transmission cycle.

Taken together, our results demonstrate the CHIKV/IRES vaccine candidate is highly and stably attenuated, and produces nearly sterilizing immunity against CHIKV challenge in the highly susceptible A129 mouse model. Together with previously reported data on preclinical safety, immunogenicity and efficacy in mice [29] and cynomolgus macaques [37], these results further support the further development of the CHIKV/IRES vaccine candidate for clinical trials.

## Supporting Information

S1 Fig A. Top view, and; B. Lateral view of E2/E1 heterodimers in trimeric spikes on the surface of chikungunya virus. The E2 envelope glycoprotein substitutions detected in the 181/clone25 vaccine strain after 5 serial passages in mice are labeled. Amino acid residues were mapped using PyMol Graphics System, Version 1.3, Schrödinger, LLC, with PDB ID 3J2W and all three occurs on the apical side of the protein in domain B that is believed to interact with cellular receptors [43]. Residue 80 is suspected to confer reversion to virulence in 181/25 p5A. Residues 82 and 57 are suspected to confer reversion to virulence in 181/25 p5B.
(JPG)

S2 Fig Full body images obtained with IVIS over a 4-day time course. The three groups represented are: Left column: CHIKV/IRES-vaccinated/CHIKV expressing firefly luciferase (CHIKV/FfLuc)-challenged; center column: sham vaccinated/CHIKV/FfLuc-challenged, and; right column: sham-vaccinated/wt-CHIKV-challenged.
(JPG)

S3 Fig Images of sham-vaccinated/CHIKV/FfLuc-challenged animals with lower extremities covered to reveal signal in other parts of the body. (JPG)

S4 Fig Viral load in multiple organs and tissues following challenge. Asterisks indicate significant differences between mock vaccine/wtCHIKV challenge and mock vaccine/CHIKV/FfLuc challenge using a student’s T-test.
(JPG)

S5 Fig Representative histopathologic examinations of the spleens in vaccinated or sham-vaccinated A129 mice three days after challenge with wt CHIKV or CHIIKV/FfLuc. A. Sham-vaccinated, sham-challenged, normal splenic architecture; B. CHIKV/IRES-vaccinated, CHIIKV/FfLuc –challenged; C. Sham-vaccinated, CHIIKV/FfLuc-challenged; D. Sham-vaccinated, wt CHIKV-challenged. Green arrow indicates proteinacious debris. Orange arrow indicates disruption of splenic architecture (remnant follicle).
(JPG)

S1 Table Primers used for RT-PCR and sequencing chikungunya virus strains after mouse passages. (DOCX)
