# Supplementary figures and images for: Extended Preclinical Safety, Efficacy and Stability Testing of a Live-attenuated Chikungunya Vaccine Candidate
Source: PLoS Negl Trop Dis. 2015 Sep 4;9(9):e0004007. doi: 10.1371/journal.pntd.0004007 (PMC4560411; doi:10.1371/journal.pntd.0004007)

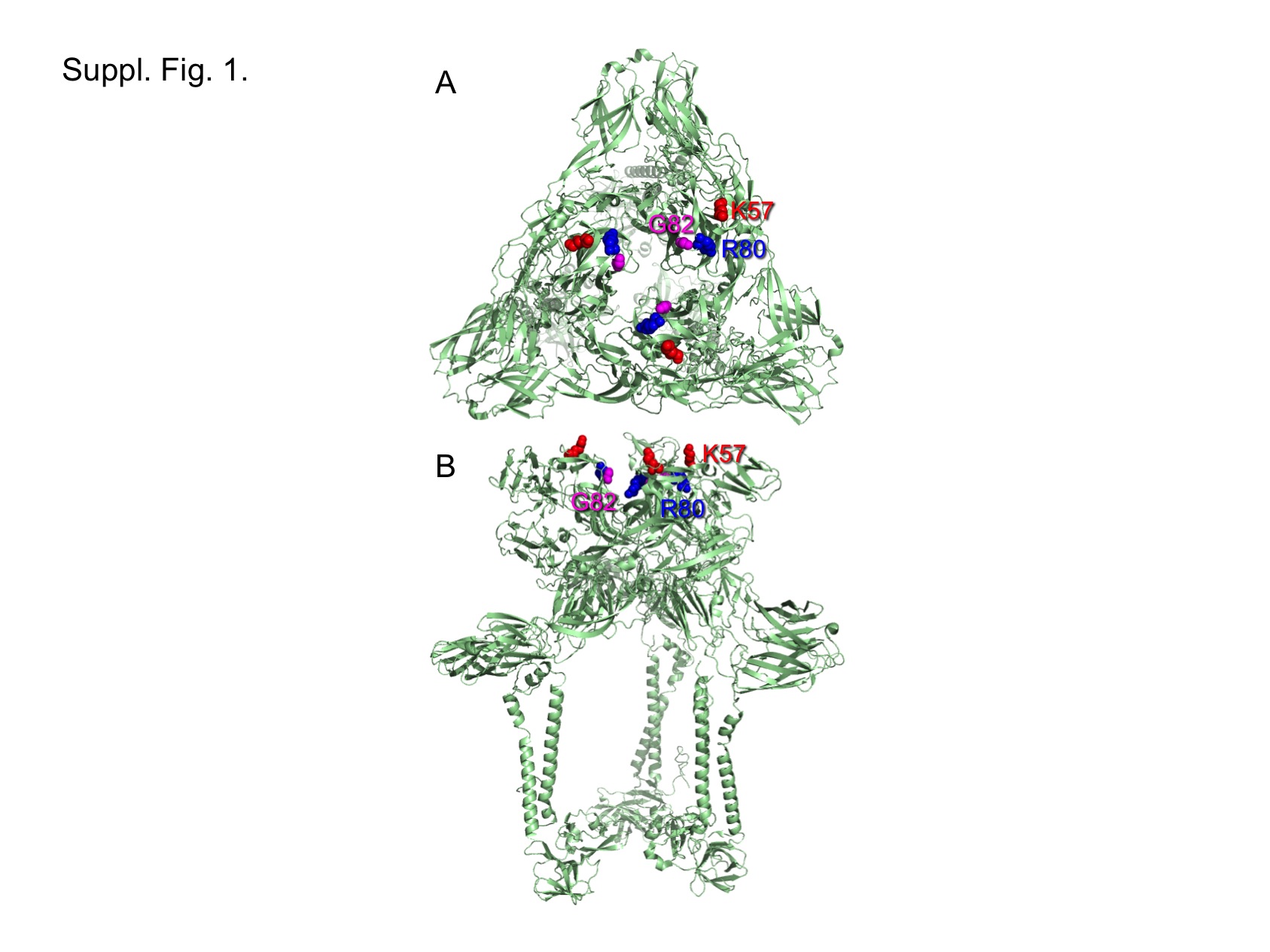

Supplement: S1 Fig — The E2 envelope glycoprotein substitutions detected in the 181/clone25 vaccine strain after 5 serial passages in mice are labeled. Amino acid residues were mapped using PyMol Graphics System, Version 1.3, Schrödinger, LLC, with PDB ID 3J2W and all three occurs on the apical side of the protein in domain B that is believed to interact with cellular receptors [43]. Residue 80 is suspected to confer reversion to virulence in 181/25 p5A. Residues 82 and 57 are suspected to confer reversion to virulence in 181/25 p5B. (JPG) [file pntd.0004007.s001.jpg]

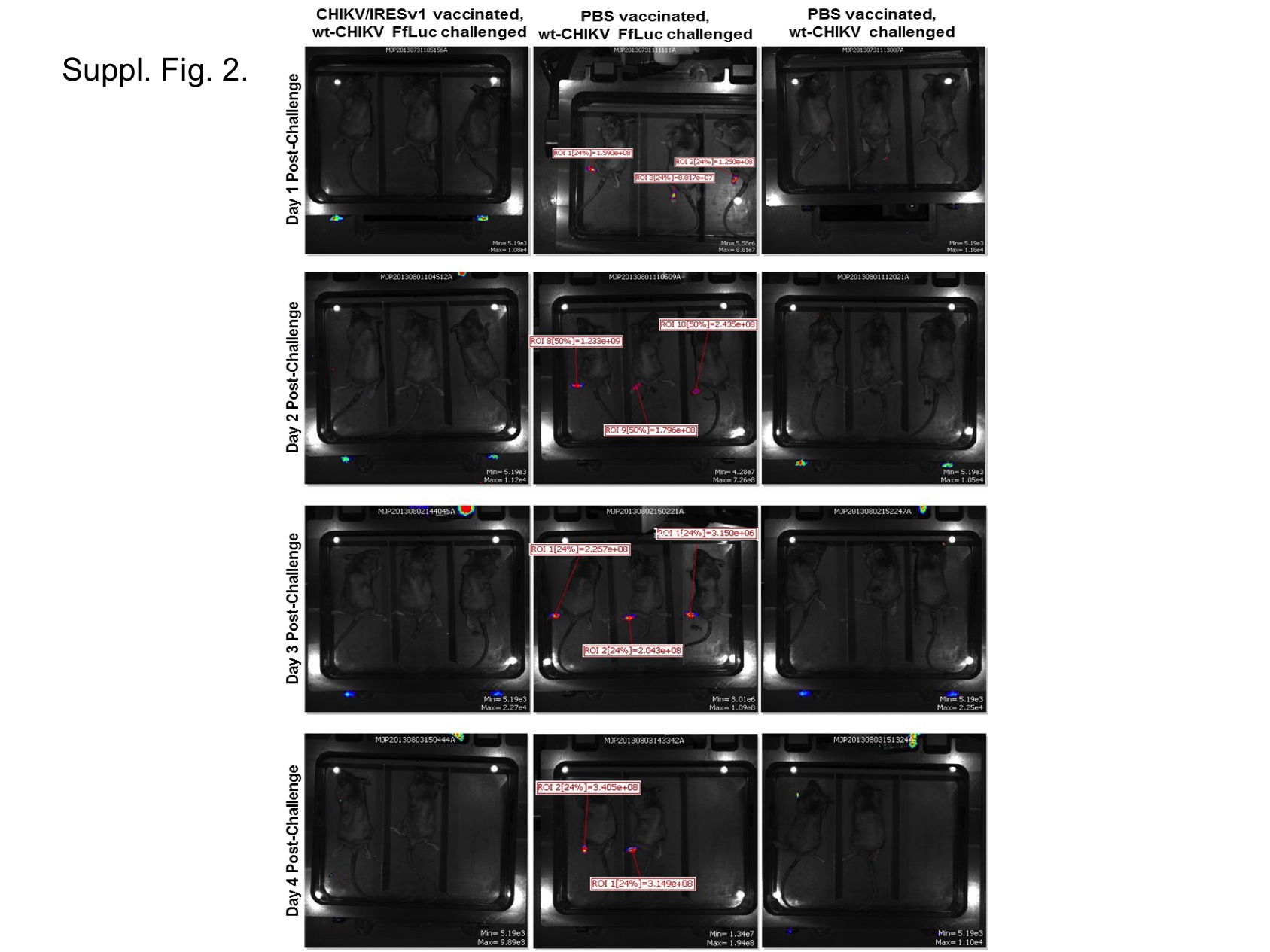

Supplement: S2 Fig — The three groups represented are: Left column: CHIKV/IRES-vaccinated/CHIKV expressing firefly luciferase (CHIKV/FfLuc)-challenged; center column: sham vaccinated/CHIKV/FfLuc-challenged, and; right column: sham-vaccinated/wt-CHIKV-challenged. (JPG) [file pntd.0004007.s002.jpg]

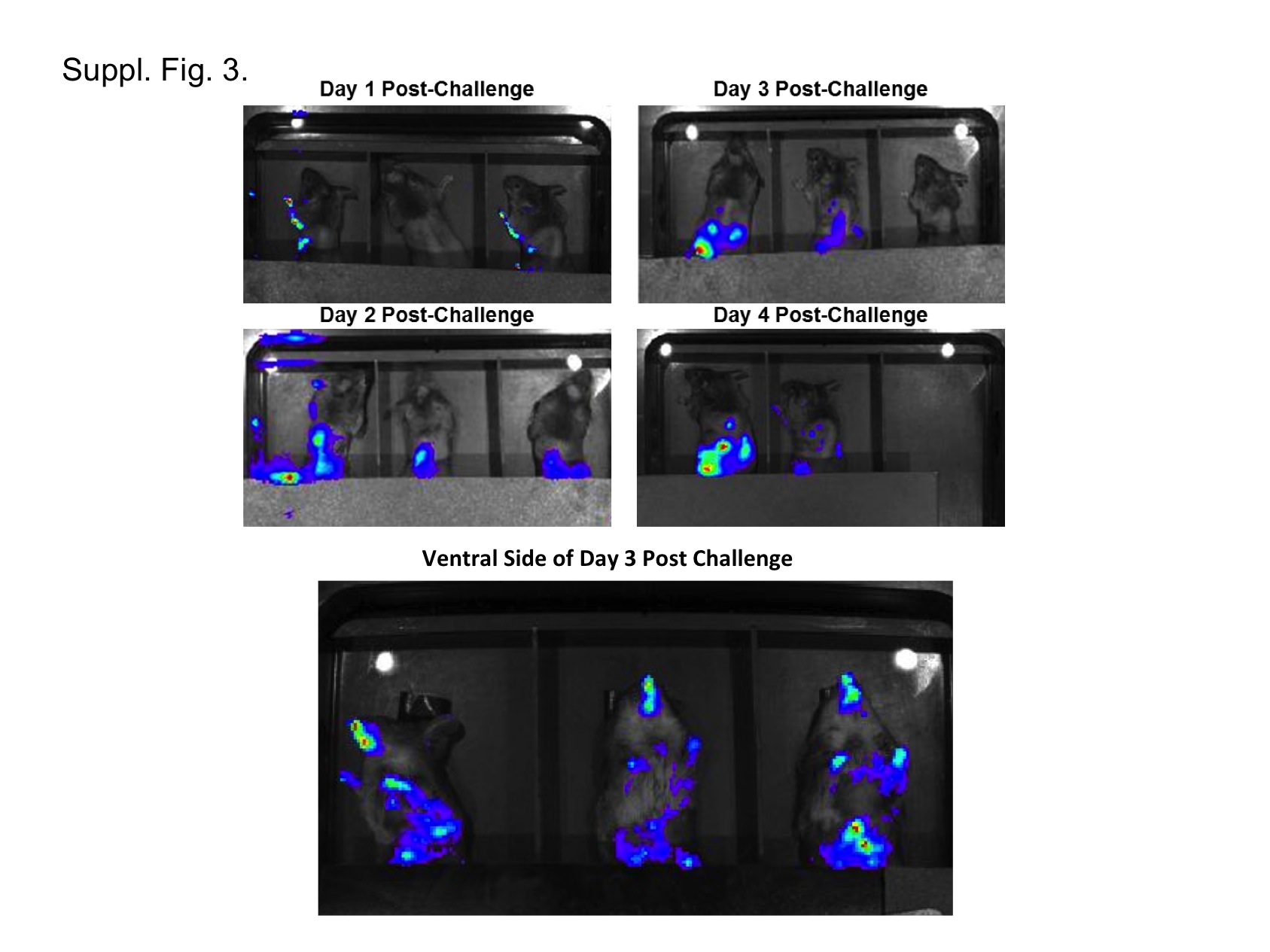

Supplement: S3 Fig — (JPG) [file pntd.0004007.s003.jpg]

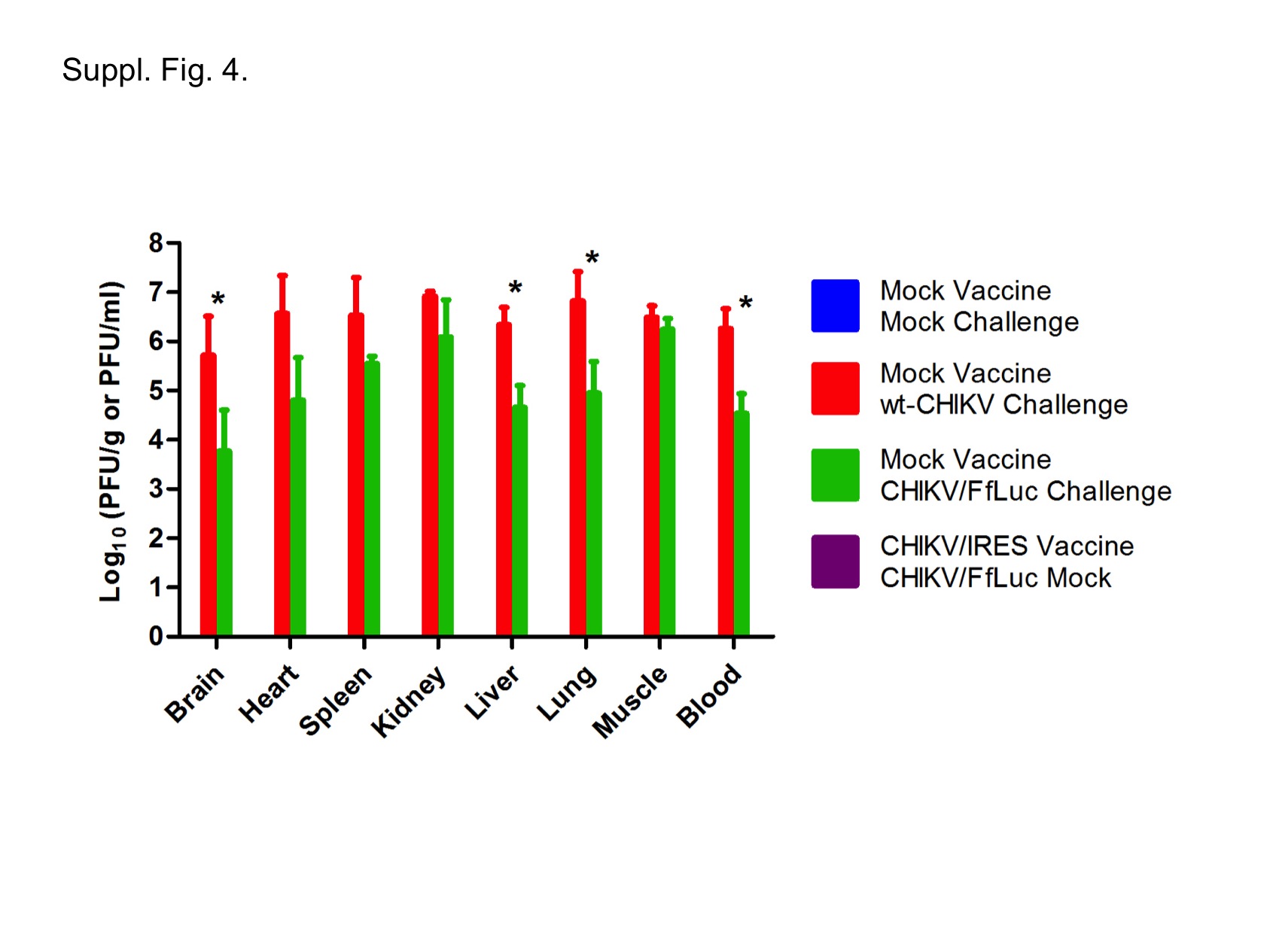

Supplement: S4 Fig — Asterisks indicate significant differences between mock vaccine/wtCHIKV challenge and mock vaccine/CHIKV/FfLuc challenge using a student’s T-test. (JPG) [file pntd.0004007.s004.jpg]

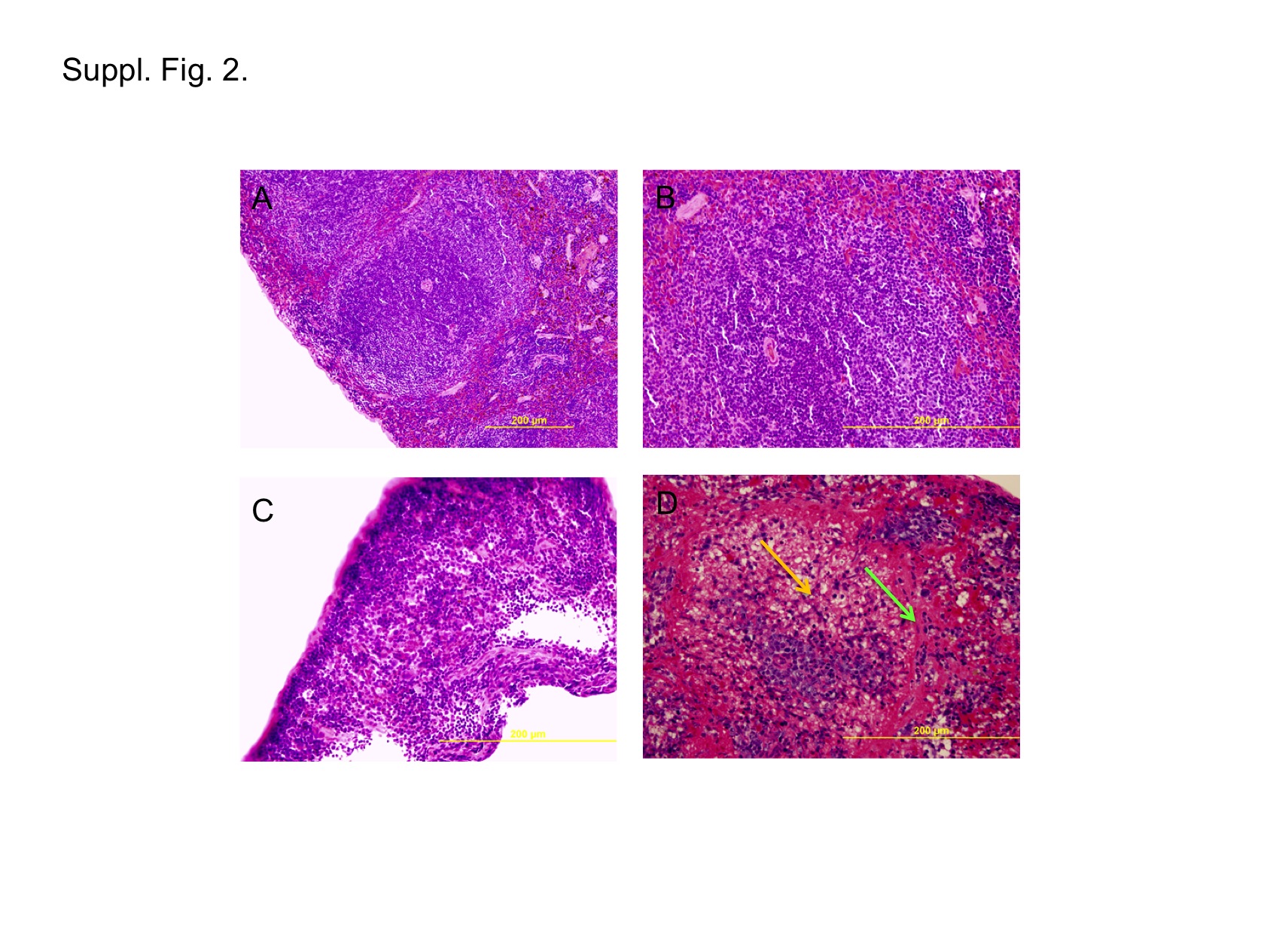

Supplement: S5 Fig — A. Sham-vaccinated, sham-challenged, normal splenic architecture; B. CHIKV/IRES-vaccinated, CHIIKV/FfLuc –challenged; C. Sham-vaccinated, CHIIKV/FfLuc-challenged; D. Sham-vaccinated, wt CHIKV-challenged. Green arrow indicates proteinacious debris. Orange arrow indicates disruption of splenic architecture (remnant follicle). (JPG) [file pntd.0004007.s005.jpg]
