# Supplementary material for: Extended Preclinical Safety, Efficacy and Stability Testing of a Live-attenuated Chikungunya Vaccine Candidate
Source: PLoS Negl Trop Dis. 2015 Sep 4;9(9):e0004007. doi: 10.1371/journal.pntd.0004007 (PMC4560411; doi:10.1371/journal.pntd.0004007)
Supplement: S1 Table — (DOCX) [file pntd.0004007.s006.docx]

| Supplemental Table 1. Primers used for RT-PCR and sequencing chikungunya virus strains after mouse passages. | |
| --- | --- |
| Primer name | Sequence (5’-3’) |
| CH2(-) 635 | GCGTTGTACATGAACGG |
| CH1(+) NEW 1 | ATGGCTGCGTGAGACAC |
| CH2(+) NEW 614 | ACAACCCCGTTCATGTA |
| CH4(+) NEW 1360 | ACACACGGTCTACAAGAGGC |
| CH5(+) NEW 1892 | GTGCCCTCAGGCTATG |
| CH6(+) NEW 2414 | ACCAGTCGACGTGTTG |
| CH7(+) NEW 3002 | GGAAATTTTAAGGCAACWATTAAGG |
| CH7(-) NEW 3200 | GGTGAGTATGCTCTGTCTTCTTT |
| CH8(+) NEW 3513 | CATTGGTGGCCGAAC |
| CH9(+) NEW 4065 | GAGCAGGGTGTGCACC |
| CH10(+) NEW 4604 | CGMGTGCACCCTGACAG |
| CH11B(+) NEW 5348 | GACGAGAGAGAAGGGAA |
| CH13(+) NEW 6114 | ACATGGTGGACGGGTC |
| CH14(+) NEW 6580 | AAGGCCTAAGGTGCAGG |
| CH15(+) NEW 7052 | TTCATCGGCGACGAC |
| CH16(-) NEW 8081 | TTCATGTGCACSGGTATCTG |
| CH17A(+) NEW 8021 | TGGCCTTTAAGCGGTC |
| CH18(+) NEW 8730 | TTGGACCAAGCTGCG |
| CH19(+) NEW 9378 | GAAAAACCAAGTCATCATGC |
| CH19(-) NEW 9419 | GTCGGATGGTCAGGATACAG |
| CH21+ 10000 | CACGTAACAGTGATCCCG |
| CHIK1133R | CAGCTTCTGTGCATCCTC |
| CHIK2528R | ATTGAAGAAGCCGCACTG |
| CHIK3498R | CTAATGAGTGTGGTAGTCTCC |
| CHIK4084R | ATGCGTTTWACCCGGTAC |
| CHIK4627R | TATCCTTTTCTGCCTGCC |
| CHIK5251R | WGGTRCGGTGYTCATTACC |
| CHIK5810R | CACTYTCCTGGAGTTTCTTAAG |
| CHIK6285R | CTCATCTGTGTGACGTTGC |
| CHIK7182R | GTGCAGTATAAACCCTCCRC |
| CHIK9037R | GGCATGTGYACCTCTATCTC |
| CHIK10574R | AAATTGTCCTGGTCTTCCTG |
| CHIK11787R | GAAATATTAAAAACAAAATAACATC |
